# Analytic center cutting plane methods for variational inequalities over convex bodies

**DOI:** 10.1186/s13660-018-1666-2

**Published:** 2018-04-14

**Authors:** Renying Zeng

**Affiliations:** 1 School of Mathematical Sciences, Chongqing Normal University, Chongqing, China; 2 Mathematics Department, Saskatchewan Polytechnic, Saskatoon, Canada

**Keywords:** 65K05, 65K10, 65K15, 90C33, Variational inequality, Quasimonotonicity, Pseudomonotonicity, Analytic center cutting plane method, Convex body

## Abstract

An analytic center cutting plane method is an iterative algorithm based on the computation of analytic centers. In this paper, we propose some analytic center cutting plane methods for solving quasimonotone or pseudomonotone variational inequalities whose domains are bounded or unbounded convex bodies.

## Introduction and preliminaries

Some recent developments in solving variational inequalities are analytic center cutting plane methods. An analytic center cutting plane method is an interior algorithm based on the computation of analytic centers. In order to work with analytic center cutting plane methods, some authors assume that the feasible sets of variational inequalities are polytopes, e.g., see [1–6], while others pay more attention to problems with infinitely many linear constraints, e.g., see [7, 8], etc. Analytic center cutting plane methods also can be used to other types of optimization problems, like mathematical programming with equilibrium constraints [9], convex programming [10, 11], conic programming [12], stochastic programming [13, 14], and combinatorial optimization [11]. In this paper, we propose some analytic center cutting plane methods for solving pseudomonotone or quasimonotone variational inequalities.

Let *X* be a nonempty subset of the *n*-dimensional Euclidean space $\mathbb{R}^{{n}}$, and let $F:X \rightarrow\mathbb{R}^{{n}}$ be a function. We say that a point $x ^{*} \in X$ is a solution of the *variational inequality* $\mathit{VI} (F, X )$ if

1 $$ F\bigl(x^{*}\bigr)^{T}\bigl(x - x^{*}\bigr) \ge 0,\quad \forall x \in X. $$

The point $x ^{*} \in X$ is a solution of the *dual variational inequality* $\mathit{VID} (F, X )$ if

2 $$ F(x)^{T}\bigl(x - x^{*}\bigr) \ge 0,\quad\forall x \in X. $$

We denote by $X ^{*}$ the set of solutions of $\mathit{VI} (F, X )$, and by $X_{D}^{*}$ the set of solutions of $\mathit{VID} (F, X )$.

From Auslender [15] we have the following lemma.

### Lemma 1

*If* *F* *is continuous*, *then a solution of* $\mathit{VID} (F, X )$ *is a solution of* $\mathit{VI} (F, X )$; *and if* *F* *is continuous pseudomonotone*, *then* $x ^{*} \in X$ *is a solution of* $\mathit{VI} (F, X )$ *if and only if it is a solution of* $\textit{VID} (F, X )$.

Given $\mathit{VI}[F, X]$ ($\textit{VID}[F, X]$), the gap function is defined as

$$\begin{gathered} g_{X}(x) = \max_{y \in X}F(x)^{T}(x - y),\quad x \in X \\ \Bigl(f_{X}(x) = \max_{y \in X}F(y)^{T}(x - y),\quad x \in X\Bigr).\end{gathered} $$

Since $g_{X}(x) \ge 0$, $f_{X}(x) \ge 0$, and

$$\begin{aligned} \arg \min_{x \in X}g_{X}(x) &= \bigl\{ g_{X}(x) = 0;x \in X \bigr\} \\ &= \arg \min_{x \in X}f_{X}(x) = \bigl\{ g_{X}(x) = 0;x \in X \bigr\} ,\end{aligned} $$

we have the following lemma.

### Lemma 2

*A point* $x ^{*} \in X$ *is a solution of* $\mathit{VI}[F,X]$ ($\mathit{VID}[F, X]$) *if and only if* $g_{X}(x^{*}) = 0$ ($f_{X}(x^{*}) = 0$).

A point $x^{*} \in X$ is said to be a *ε-solution* of the variational inequality (1) if $g_{X}(x^{*}) < \varepsilon$.

A function $F: X \rightarrow \mathbb{R}^{{n}}$ is said to be *monotone* on *X* if

$$\bigl(F(y) - F(x)\bigr)^{T}(y - x) \ge 0,\quad\forall x,y \in X; $$

*strongly monotone* if there exists a constant $M > 0$ such that

$$\bigl(F(y) - F(x)\bigr)^{T}(y - x) \ge M \Vert y - x \Vert ,\quad \forall x,y \in X; $$

*quasimonotone* on *X* if

$$F(x)^{T}(y - x) > 0\quad \Rightarrow \quad F(y)^{T}(y - x) \ge 0,\quad \forall x,y \in X; $$

*pseudomonotone* on *X* if

$$F(x)^{T}(y - x) \ge 0 \quad\Rightarrow\quad F(y)^{T}(y - x) \ge 0,\quad \forall x,y \in X; $$

*pseudomonotone* *plus* on *X* if it is pseudomonotone on *X* and if

$$\left . \textstyle\begin{array}{c} F(x)^{T}(y - x) \ge 0 \\ F(y)^{T}(y - x) = 0 \end{array}\displaystyle \right \} \quad\Rightarrow\quad F(x) = F(y),\quad\forall x,y \in X; $$

and *strongly* *pseudomonotone* on *X* if there exist constants $M > 0$, $\alpha > 0$ such that

$$F(x)^{T}(y - x) \ge 0 \quad\Rightarrow\quad F(y)^{T}(y - x) \ge M \Vert y - x \Vert ^{\alpha},\quad\forall x,y \in X. $$

## Results and discussion

We proposed some analytic center cutting plane methods (ACCPM) for convex feasibility problems. Convex feasibility problem is a problem of finding a point in a convex set, which contains a full dimensional ball and is contained in a compact convex set described by matrix inequalities. There are many applications of these types of problems in nonsmooth optimization. The ACCPM is an efficient technique for nondifferentiable optimization. We employed some nonpolyhedral models into the ACCPM.

We present five analytic center cutting plane methods for solving variational inequalities whose domains are bounded or unbounded convex bodies.

First four algorithms are for the variational inequalities with compact and convex feasible sets. If $F: X \rightarrow\mathbb{R}^{{n}}$ is pseudomonotone plus on a compact convex body *X*, then our Algorithm 1 either stops with a solution of the variational inequality $\mathit{VI} (F, X )$ after a finite number of iterations, or there exists an infinite sequence $\{x_{j}\}$ in *X* that converges to a solution of $\mathit{VI} (F, X )$. If $F: X \rightarrow\mathbb{R}^{{n}}$ is pseudomonotone plus on a compact convex body *X*, then our Algorithm 2 stops with an *ε*-solution of the variational inequality $\mathit{VI} (F, X )$ after a finite number of iterations. If $F: X \rightarrow\mathbb{R}^{{n}}$ is Lipschitz continuous on a compact convex body *X*, then our Algorithm 3 either stops with a solution of the variational inequality $\mathit{VI} (F, X )$ after a finite number of iterations, or there exists an infinite sequence $\{x_{j}\}$ in *X* that converges to a solution of $\mathit{VI} (F, X )$. If $F: X \rightarrow\mathbb{R}^{{n}}$ is Lipschitz continuous on a compact convex body *X*, then our Algorithm 4 stops with an *ε*-solution of $\mathit{VI} (F, X )$ after a finite number of iterations.

Our fifth algorithm is for variational inequalities with unbounded compact convex feasible regions, and these feasible regions can be the *n*-dimensional Euclidean space $\mathbb{R}^{{n}}$ itself. If $F: X \rightarrow\mathbb{R}^{{n}}$ is strongly monotone on *X*, then our Algorithm 5 either stops with a solution of the variational inequality $\mathit{VI} (F, X )$ after a finite number of iterations, or there exists an infinite sequence $\{x_{j}^{k}\}$ in *X* that converges to a solution of $\mathit{VI} (F, X )$. Furthermore, the proof of the previous result also indicates that, if $F: X \rightarrow\mathbb{R}^{{n}}$ is strongly pseudomonotone on *X*, then our Algorithm 5 either stops with a solution of $\mathit{VI} (F, X )$ after a finite number of iterations, or there exists an infinite sequence $\{x_{j}\}$ in *X* that converges to a solution of $\mathit{VI} (F, X )$.

## Conclusions

This paper works with variational inequalities whose feasible sets are bounded or unbounded convex bodies. We present some analytic center cutting plane algorithms that extend the algorithms proposed in [1, 2, 16], from polytopes/polyhedron to convex regions, or from bounded convex region to unbounded convex regions. We should mention that our approach can be used to extend many interior methods which are associated with polyhedral feasible regions, e.g., the algorithms given by [3, 4]. We can also extend some other algorithms for variational inequalities over polyhedral feasible sets [17–19].

## Compact convex bodies

A *polytope* is a set $P \subseteq\mathbb{R}^{{n}}$ which is the convex hull of a finite set.

A *polyhedron* is a set

$$\bigl\{ x \in \mathbb{R}^{n};A^{T}x \le b\bigr\} \subseteq \mathbb{R}^{{n}}, $$

where $b \in\mathbb{R}^{{n}}$, and *A* is an $m \times n$ matrix.

Every polytope is a polyhedron, whereas not every polyhedron is a polytope.

Minkowski proved the following lemma in 1896.

### Lemma 3

*A set* $P \subseteq\mathbb{R}^{{n}}$ *is a polytope if and only if it is a bounded polyhedron*.

We make the following assumptions for polytopes throughout this paper.

(a) *Interior assumption*: A polytope is always a full-dimensional polytope and that includes $0 \le x \le e$, where *e* is a vector of all ones.

We note that if a polytope has nonempty interior, then (a) can be met by re-scaling.

A *convex body* $X \subseteq\mathbb{R}^{{n}}$ is a convex and bounded subset with nonempty interior.

A *rectangle* $B \subseteq\mathbb{R}^{{n}}$ is defined by

$$B =\bigl\{ x = (x_{1},x_{2}, \ldots,x_{n}) \in \mathbb{R}^{n};a_{i} \le x_{i} \le b_{i}\bigr\} , $$

where $a _{i}, b _{i} \in\mathbb{R}$.

A rectangle can also be given by some inequalities

$$B =\bigl\{ x \in \mathbb{R}^{n};H^{T}x \le b\bigr\} , $$

where $H^{T}x = b$ is a finite set of hyperplanes, *H* is an $m \times n$ matrix. And, if we denote by *V* the finite set of all vertices of *B*, then

$$B = \operatorname{con} (V). $$

### Theorem 1

*A bounded subset* $X \subseteq\mathbb{R}^{{n}}$ *is a compact convex body if and only if there exists a sequence of polytopes* $\{C _{j}\}$ *satisfying* $C _{j} \subseteq C_{j + 1}$ ($j = 1, \ldots$) *such that*

$$\Biggl(\bigcup_{j = 1}^{\infty} C_{j} \Biggr)^{c} = X. $$

### Proof

The sufficiency is trivial. We only prove the necessity.

Since *X* is bounded, there exists a rectangle *B* such that $X \subseteq B$.

**Figure 1:**
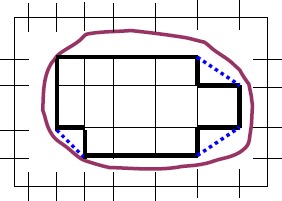
$C _{1} = \operatorname{con}(V _{1})$

Take a partition $P _{1}$ of *B*. Then *B* is divided into a set of finite sub-rectangles by a finite set of hyperplanes. Let $D _{1} = \bigcup_{j = 1}^{k_{1}} B_{1(j)}$, where $B_{1(j)}$ ($j = 1, \dots, k _{1}$) are all the sub-rectangles that lie entirely within *X*. Let $V _{1}$ be the set of all vertices of $B_{1(j)}$ ($j = 1, \dots, k _{1}$), then $V _{1}$ is a finite set. So, $C _{1} = \operatorname{con}(V _{1})$ is a polytope, and it obviously satisfies

$$D_{1} \subseteq C_{1} \subseteq X. $$

(For the case of a 2-dimensional Euclidean space, see Fig. 1.)

Take a finer partition $P _{2}$ of *B*. Similarly, we have a set $D _{2} = \bigcup_{j = 1}^{k_{2}} B_{2(j)}$, where $B_{2(j)}$ ($j = 1, \dots, k _{2}$) are all the sub-rectangles which correspond to $P _{2}$ and lie entirely within *X*; and we have a polytope $C _{2} = \operatorname{con}(V _{2})$, where $V _{2}$ is the set of all vertices of $B_{2(j)}$ ($j = 1, \dots, k _{2}$) such that

$$C_{1} \subseteq C_{2} \subseteq X. $$

By mathematical induction, there exists a sequence of polytopes $\{C_{j}\}$ which satisfies

$$C _{j} \subseteq C_{j + 1} \subseteq X\quad (j = 1, \ldots). $$

It is easy to see that $(\bigcup_{j = 1}^{\infty} C_{j} )^{c} = X$. □

It is quite straightforward to prove the following Corollary 1, Proposition 1, and Proposition 2.

### Corollary 1

*A subset* $X \subseteq\mathbb{R}^{{n}}$ *is a compact convex body if there is a uniformly bounded sequence of polytopes* $\{C_{j}\}$, *i*.*e*., $C _{j} \subseteq B $ *for a given rectangle* *B*, *such that*

$$\Biggl(\bigcup_{j = 1}^{\infty} C_{j} \Biggr)^{c} = X. $$

### Proposition 1

*Let* $X \subseteq\mathbb{R}^{{n}}$ *be a compact convex body and* $F: X \rightarrow\mathbb{R}^{{n}}$ *be a continuous function*, *then the variational inequality* $\mathit{VI}[F, X]$ *has solutions*.

### Proposition 2

*Let* $X \subseteq\mathbb{R}^{{n}}$ *be a compact convex body and* $F:X \rightarrow\mathbb{R}^{{n}}$ *be a continuous and strictly pseudomonotone function*, *then the variational inequality* $\mathit{VI}[F, X]$ *has a unique solution*.

## Generalized analytic center cutting plane algorithms for solving pseudomonotone variational inequalities

For any polytope $\{ x \in \mathbb{R}^{n};A^{T}x \le b\}$,

$$\bigl\{ x \in {R}^{n};A^{T}x + s = b,s = (s_{1},s_{2}, \ldots,s_{n}),s_{i} \ge 0\bigr\} $$

is associated with the potential function

$$\varphi = \sum_{i = 1}^{n} \ln s_{i}. $$

It is known that an *analytic center* is the maximizer of the potential function *φ*, and the unique solution of the system

$$\begin{gathered} A^{T}y = 0, \\ A^{T}x + s = b, \\ Y^{T}s = e, \end{gathered} $$

where *y* is a positive dual vector, and *Y* the diagonal matrix built upon *y*.

An *approximate analytic center* [20] is the maximizer of the potential function *φ*, and the unique solution of the system

$$\begin{gathered} A^{T}z = 0, \\ A^{T}x + s = b, \\ \big\| Z^{T}s - e\big\| \le \eta < 1, \end{gathered} $$

where *z* is a dual vector, and *Z* is the diagonal matrix built upon *z*.

Now we modify Goffin, Marcotte, and Zhu’s [2] Algorithm 1 to solve $\mathit{VI} (F, X )$. We propose an algorithm for solving variational inequalities, whose feasible sets are compact convex bodies.

From Theorem 1, there exists a sequence of variational inequalities $\mathit{VI} [F, C _{j}]$ ($j = 1, \ldots$) induced by the original variational inequality $\mathit{VI}[F, X]$, where the polytope $C _{j}$ is given by the linear inequalities $A_{j}^{T}x = b_{j}$, $x, b _{j} \in\mathbb{R}^{{n}}$, and $A _{j}$ is an $m \times n$ matrix. So, we may apply the algorithm in [2] to each $\mathit{VI} [F, C _{j}]$. Algorithm 1 uses this idea, but the algorithm in [2] is applied to $\mathit{VI} [F, C _{j}]$ for only a certain number of iterations until we get

$$g_{C_{j}}\bigl(x_{j}^{k}\bigr) < \frac{1}{2^{j}}\quad (j = 1, \ldots), $$

by use of Theorem 1 of [2].

### Algorithm 1

- *Step 1*. (initialization)
  $$k = 0, \qquad j = 1,\qquad A^{k} = A_{j},\qquad b^{k} = b_{j},\qquad C_{j}^{k} = \bigl\{ x \in \mathbb{R}^{n};A_{j}^{k}x \le b_{j}^{k} \bigr\} ; $$
- *Step 2*. (computation of an approximate analytic center)
  Find an approximate analytic center $x_{j}^{k}$ of $C_{j}^{k}$;
- *Step 3*. (stop criterion)
  Compute $g_{X}(x_{j}^{k}) = \max_{x \in X}F(x_{j}^{k})^{T}(x_{j}^{k} - x)$,
  if $g_{X}(x_{j}^{k}) = 0$, **then** STOP,
  **else** GO TO step 4;
- *Step 4*. (find an *ε*-solution for $\varepsilon = \frac{1}{2^{j}}$)
  Compute $g_{C_{j}}(x_{j}^{k}) = \max_{x \in C_{j}}F(x_{j}^{k})^{T}(x_{j}^{k} - x)$,
  if $g_{C_{j}}(x_{j}^{k}) < \frac{1}{2^{j}}$, **then** increase *j* by one RETURN TO Step 1,
  **else** GO TO Step 5;
- *Step 5*. (cut generation)
  Set
  $$A_{j}^{k + 1} = \left [ \textstyle\begin{array}{l} A_{j}^{k} \\ F(x_{j}^{k})^{T} \end{array}\displaystyle \right ],\qquad b_{j}^{k + 1} = \left [ \textstyle\begin{array}{l} b_{j}^{k} \\ F(x_{j}^{k})^{T}x_{j}^{k} \end{array}\displaystyle \right ], $$
  $H_{j}^{k} = \{ x \in \mathbb{R}^{n};F(x_{j}^{k})^{T}(x - x_{j}^{k}) = 0\}$ is the new cutting plane for $\mathit{VI}(F, C_{j}^{k})$.
  Increase *k* by one GO TO Step 2.

### Theorem 2

*Let* $F: X \rightarrow\mathbb{R}^{{n}}$ *be pseudomonotone plus on a compact convex body* *X*, *then Algorithm *1 *either stops with a solution of* $\mathit{VI} (F, X )$ *after a finite number of iterations*, *or there exists a subsequence of the infinite sequence* $\{x_{j}^{k}\}$ *that converges to a point* $x ^{*} \in X ^{*}$.

### Proof

According to Algorithm 1 and Theorem 1 of [2], for any given *j*, $\exists x _{j} \in C _{j}$ such that after a finite number of iterations,

$$g_{C_{j}}(x_{j}) < \frac{1}{2^{j}}. $$

Since *X* is compact, there exists a subsequence $\{x _{j (q)}\}$ of $\{x _{j} \}$ and a point $x ^{*} \in X$ such that

$$\lim_{q \to \infty} x_{j(q)} = x^{*}. $$

$\forall p < j$, we have

$$g_{C_{p}}(x_{j}) \le g_{C_{j}}(x_{j}) < \frac{1}{2^{j}}. $$

On the other hand, due to the compactness of *X*, $\exists N > 0$ such that $\| y \| \leq N$, $\forall y \in X$. Since

$$\max_{y \in X}F\bigl(x'\bigr)^{T} \bigl(x' - y\bigr) \ge 0\quad \mbox{and}\quad \max _{y \in X}F\bigl(x''\bigr)^{T} \bigl(x'' - y\bigr) \ge 0, $$

for $\forall x',x'' \in X$,

$$\begin{gathered} \big|g_{X}\bigl(x'\bigr) - g_{X}\bigl(x''\bigr)\big| \\ \quad= \Big|\max_{y \in X}F\bigl(x'\bigr)^{T} \bigl(x' - y\bigr) - \max_{y \in X}F \bigl(x''\bigr)^{T}\bigl(x'' - y\bigr)\Big| \\ \quad\le \max_{y \in X}\big|F\bigl(x' \bigr)^{T}\bigl(x' - y\bigr) - F\bigl(x'' \bigr)^{T}\bigl(x'' - y\bigr)\big| \\ \quad= \max_{y \in X}\big|\bigl[F\bigl(x'\bigr)^{T}x' - F\bigl(x''\bigr)^{T}x'' \bigr] + \bigl[F\bigl(x''\bigr)^{T}y - F \bigl(x'\bigr)^{T}y\bigr]\big| \\ \quad\le\big|F\bigl(x'\bigr)^{T}x' - F \bigl(x''\bigr)^{T}x''\big| + \max_{y \in X}\big|F\bigl(x'' \bigr)^{T}y - F\bigl(x'\bigr)^{T}y\big| \\ \quad\le \big|F\bigl(x'\bigr)^{T}x' - F \bigl(x''\bigr)^{T}x''\big| + N\big\| F\bigl(x''\bigr) - F\bigl(x'\bigr) \big\| . \end{gathered} $$

By the continuities of $F(x)$ and $F(x)^{T} x$, $g_{X}(x)$ is a continuous function on *X*.

Consequently, ∀*p*

$$g_{C_{p}}\bigl(x^{*}\bigr) = \lim_{q \to \infty} g_{C_{p}}(x_{j(q)}) \le \lim_{q \to \infty} g_{C_{j(q)}}(x_{j(q)}) \le \lim_{q \to \infty} \frac{1}{2^{j(q)}} = 0. $$

Then we have

$$\begin{aligned} g_{\bigcup_{j = 1}^{\infty} C_{j}} \bigl(x^{*}\bigr)& = \max_{y \in \bigcup_{j = 1}^{\infty} C_{j}} F\bigl(x^{*}\bigr)\bigl(x^{*} - y\bigr) \\ &\le \sum_{j = 1}^{\infty} \max_{y \in C_{j}}F\bigl(x^{*}\bigr)\bigl(x^{*} - y\bigr) = \sum_{j = 1}^{\infty} g_{C_{j}}\bigl(x^{*}\bigr) = 0. \end{aligned} $$

On the other hand,$\forall y \in X$, $\exists \{ y_{i}\} \subseteq \bigcup_{j = 1}^{\infty} C_{j}$ such that

$$\lim_{i \to \infty} y_{i} = y. $$

Because

$$\begin{aligned} \big|F\bigl(x^{*}\bigr)\bigl(x^{*} - y_{i}\bigr)\big| &\le \max_{y \in \bigcup_{j = 1}^{\infty} C_{j}} F\bigl(x^{*}\bigr)\bigl(x^{*} - y\bigr) \\ &= g_{\bigcup_{j = 1}^{\infty} C_{j}} \bigl(x^{*}\bigr) = 0, \end{aligned} $$

we have

$$\big|F\bigl(x^{*}\bigr) \bigl(x^{*} - y\bigr)\big| = \lim _{i \to \infty} \big|F\bigl(x^{*}\bigr) \bigl(x^{*} - y_{i}\bigr)\big| = 0. $$

Therefore,

$$g_{X}\bigl(x^{*}\bigr) = \max_{y \in X}F \bigl(x^{*}\bigr) \bigl(x^{*} - y\bigr) = 0, $$

which deduces that $x^{*}$ is a solution of $\mathit{VI} (F, X )$. □

Algorithm 1 usually generates an infinite sequence. In order to terminate at a finite number of iterations, we change the stop criterion, Step 3 in Algorithm 1, to get the following algorithm.

### Algorithm 2

Step 1, Step 2, Step 4, and Step 5 are the same as those of Algorithm 1.

- *Step 3*. (stop criterion)
  Compute $g_{X}(x_{j}^{k}) = \max_{x \in X}F(x_{j}^{k})^{T}(x_{j}^{k} - x)$,
  if $g_{X}(x_{j}^{k}) < \varepsilon$, **then** STOP,
  **else** GO TO step 4.

From Theorem 2 we have the following.

### Theorem 3

*Let* $F: X \rightarrow\mathbb{R}^{{n}}$ *be pseudomonotone plus on a compact convex body* *X*, *then Algorithm *2 *stops with an* *ε*-*solution of* $\mathit{VI} (F, X )$ *after a finite number of iterations*.

## Generalized analytic center cutting plane algorithms for solving quasimonotone variational inequalities

In this section, we are going to modify Marcotte and Zhu’s [1] approach to solve quasimonotone variational inequalities $\mathit{VI} (F, X )$. We assume that the feasible sets are compact convex bodies.

From Theorem 1 there is a sequence of variational inequalities $\mathit{VI} [F, C_{{j}}]$ ($j = 1, \ldots$) induced by the original variational inequality $\mathit{VI}[F, X]$.

According to [1], the following are the conditions that are required in the construction of algorithms for solving quasimonotone variational inequalities.

For any given *j*, let the auxiliary function $\Gamma_{j}(y,x):\mathbb{R}^{n} \to \mathbb{R}^{n}$ be continuous in *x* and strongly monotone in *y*, i.e.,

$$\bigl\langle \Gamma_{j}\bigl(y',x\bigr) - \Gamma_{j}\bigl(y'',x\bigr),y' - y'' \bigr\rangle \ge \beta_{j} \big\| y' - y''\big\| ^{2},\quad\forall y',y'' \in X $$

for $\beta_{j} > 0$. $\beta_{j}$ is said to be the strong monotonicity constant for $\Gamma_{j}(y,x):\mathbb{R}^{n} \to \mathbb{R}^{n}$. The function $\Gamma _{j}$ is associated with the variational inequality $\textit{AVI}[\Gamma, X, x]$ whose solution $w _{j}(x)$ satisfies

$$\bigl\langle \Gamma_{j}\bigl(w(x),x\bigr) - \Gamma_{j}(x,x) + F(x),y - w_{j}(x) \bigr\rangle \ge 0,\quad\forall y \in X. $$

It is known that $w _{j}(x)$ are continuous [21], and that *x* is a solution of $\mathit{VI}[F,C _{j}]$ if and only if it is a fixed point of *w*.

Assume $0 < \rho _{j} < 1$ and $0 < \alpha _{j} < \beta _{j}$. Let $l(j)$ (which depends on *x*) be the smallest nonnegative integer for which

$$\bigl\langle F\bigl(x + \rho_{j}^{l(j)}\bigl(w_{j}(x) - x\bigr)\bigr),x - w_{j}(x) \bigr\rangle \ge \alpha_{j} \big\| w_{j}(x) - x_{j}\big\| ^{2}. $$

Define

$$G_{j}(x) = F\bigl(x + \rho_{j}^{l(j)} \bigl(w_{j}(x) - x\bigr)\bigr). $$

If $x_{j}^{*}$ is a solution of $\mathit{VI}[F,C _{j}]$, then $w_{j}(x_{j}^{*}) = x_{j}^{*}$, $l(j) = 0$, and $G_{j}(x_{j}^{*}) = F(x_{j}^{*})$.

### Algorithm 3

- *Step 1*. (initialization)
  Let $\beta_{j} > 0$ be the strong monotonicity constant for $\Gamma_{j}(y,x):\mathbb{R}^{n} \to \mathbb{R}^{n}$, with respect to *y*, and let $\alpha _{j} \in (0, \beta_{j})$.
  $$k = 0,\qquad j = 1,\qquad A^{k} = A_{j},\qquad b^{k} = b_{j},\qquad C_{j}^{k} = \bigl\{ x \in \mathbb{R}^{n};A_{j}^{k}x \le b_{j}^{k} \bigr\} ; $$
- *Step 2*. (computation of an approximate analytic center)
  Find an approximate analytic center $x_{j}^{k}$ of $C_{j}^{k}$;
- *Step 3*. (stop criterion)
  Compute $g_{X}(x_{j}^{k}) = \max_{x \in X}F(x_{j}^{k})^{T}(x_{j}^{k} - x)$,
  if $g_{X}(x_{j}^{k}) = 0$, **then** STOP,
  **else** GO TO step 4;
- *Step 4*. (find an *ε*-solution for $\varepsilon = \frac{1}{2^{j}}$)
  Compute $g_{C_{j}}(x_{j}^{k}) = \max_{x \in C_{j}}F(x_{j}^{k})^{T}(x_{j}^{k} - x)$,
  if $g_{C_{j}}(x_{j}^{k}) < \frac{1}{2^{j}}$, **then** increase *j* by one RETURN TO Step 1,
  **else** GO TO Step 5;
- *Step 5*. (auxiliary variational inequality)
  Let $w_{j}(x_{j}^{k})$ satisfy the variational inequality
  $$\bigl\langle F\bigl(x_{j}^{k}\bigr) + \Gamma_{j} \bigl(w_{j}\bigl(x_{j}^{k}\bigr),x_{j}^{k} \bigr) - \Gamma_{j}\bigl(x_{j}^{k},x_{j}^{k} \bigr),y - w_{j}\bigl(x_{j}^{k}\bigr) \bigr\rangle \ge 0,\quad\forall y \in C_{j}. $$
  Let
  $$y_{j}^{k} = x_{j}^{k} + \rho_{j}^{l(k,j)}\bigl(w_{j}\bigl(x_{j}^{k} \bigr) - x_{j}^{k}\bigr) \quad \mbox{and}\quad G_{j} \bigl(x_{j}^{k}\bigr) = F\bigl(y_{j}^{k} \bigr), $$
  where $l(k, j)$ is the smallest integer which satisfies
  $$\bigl\langle F\bigl(x_{j}^{k} + \rho_{j}^{l(k,j)} \bigl(w_{j}\bigl(x_{j}^{k}\bigr) - x_{j}^{k}\bigr)\bigr),x_{j}^{k} - w_{j}\bigl(x_{j}^{k}\bigr) \bigr\rangle \ge \alpha_{j}\big\| w_{j}\bigl(x_{j}^{k}\bigr) - x_{j}^{k}\big\| ^{2}; $$
- *Step 6*. (cutting plane generation)
  Set
  $$A_{j}^{k + 1} = \left [ \textstyle\begin{array}{l} A_{j}^{k} \\ G(x_{j}^{k})^{T} \end{array}\displaystyle \right ],\qquad b_{j}^{k + 1} = \left [ \textstyle\begin{array}{l} b_{j}^{k} \\ G(x_{j}^{k})^{T}x_{j}^{k} \end{array}\displaystyle \right ], $$
  $H_{j}^{k} = \{ x \in \mathbb{R}^{n};G(x_{j}^{k})^{T}(x - x_{j}^{k}) = 0\}$ is the new cutting plane for $\mathit{VI}(F, C_{j}^{k})$.
  Increase *k* by one GO TO Step 2.

By Theorem 1 of [1], similar to the proof of Theorem 2, we have the following theorem.

### Theorem 4

*Let* $F: X \rightarrow\mathbb{R}^{{n}}$ *be Lipschitz continuous*, *i*.*e*., *there exists a constant* $L > 0$ *such that*

$$\bigl(F(y) - F(x)\bigr)^{T}(y - x) \le L \Vert y - x \Vert ,\quad \forall x,y \in X $$

*on a compact convex body* *X*, *and* $X_{D}^{*}$ *be nonempty*. *Then Algorithm *3 *either stops with a solution of* $\mathit{VI} (F, X )$ *after a finite number of iterations*, *or there exists a subsequence of the infinite sequence* $\{x_{j}^{k}\}$ *that converges to a point* $x ^{*} \in X ^{*}$.

### Algorithm 4

Step 1, Step 2, Step 4, Step 5, and Step 6 are the same as those in Algorithm 3.

- *Step 3*. (stop criterion)
  Compute $g_{X}(x_{j}^{k}) = \max_{x \in X}F(x_{j}^{k})^{T}(x_{j}^{k} - x)$,
  if $g_{X}(x_{j}^{k}) < \varepsilon$, **then** STOP,
  **else** GO TO step 4.

By Theorem 4 we have the following.

### Theorem 5

*Let* $F: X \rightarrow\mathbb{R}^{{n}}$ *be Lipschitz continuous on a compact convex body* *X* *and* $X_{D}^{*}$ *be nonempty*. *Then Algorithm *4 *stops with an* *ε*-*solution of* $\mathit{VI} (F, X )$ *after a finite number of iterations*.

## Generalized analytic center cutting plane algorithms for variational inequalities with unbounded domains

This section presents analytic center cutting plane algorithms for solving a strongly pseudomonotone variational inequality $\mathit{VI}[F, X]$ whose domain is an unbounded convex body. By use of Propositions 1 and 2, due to the pseudomonotonicity, $\mathit{VI}[F, X]$ has a unique solution $x ^{*}$ over *X*. Let $\{C _{j}\}$ be a sequence of polytopes that satisfies

$$C _{j} \subseteq C_{j + 1}\quad (j = 1, \ldots),\quad \text{and}\quad \Biggl(\bigcup_{j = 1}^{\infty} C_{j} \Biggr)^{c} = \mathbb{X}, $$

then $\mathit{VI}[F, C _{j}]$ has a unique solution $x_{j}^{*}$ over $C _{j}$ ($j = 1, 2, \dots$). We can always assume that $C _{j}$ contains all boundary points of *X* (if there are any). Since the solution $x ^{*}$ of $\mathit{VI}[F, X]$ is a fixed point, $x ^{*}$ lies in $C _{j}$ if *j* is large enough (say $j > k$), therefore by Lemma 2 $x_{j}^{*}= x ^{*}$ ($j > k$).

The following algorithm is proposed here to find $x ^{*}$.

### Algorithm 5

- *Step 1*. (initialization)
  $$k = 0,\qquad j = 1,\qquad A^{k} = A_{j},\qquad b^{k} = b_{j},\qquad C_{j}^{k} = \bigl\{ x \in \mathbb{R}^{n};A_{j}^{k}x \le b_{j}^{k} \bigr\} ; $$
- *Step 2*. (find an *ε*-solution for $\varepsilon = \frac{1}{2^{j}}$)
  Find an approximate analytic center $x_{j}^{k}$ of $C_{j}^{k}$.
  Compute $g_{C_{j}}(x_{j}^{k}) = \max_{x \in C_{j}}F(x_{j}^{k})^{T}(x_{j}^{k} - x)$,
  if $g_{C_{j}}(x_{j}^{k}) < \frac{1}{2^{j}}$, **then** increase *j* by one RETURN TO Step 1,
  **else** GO TO Step 3;
- *Step 3*. (cut generation)
  Set
  $$A_{j}^{k + 1} = \left [ \textstyle\begin{array}{l} A_{j}^{k} \\ F(x_{j}^{k})^{T} \end{array}\displaystyle \right ],\qquad b_{j}^{k + 1} = \left [ \textstyle\begin{array}{l} b_{j}^{k} \\ F(x_{j}^{k})^{T}x_{j}^{k} \end{array}\displaystyle \right ], $$
  $H_{j}^{k} = \{ x \in \mathbb{R}^{n};F(x_{j}^{k})^{T}(x - x_{j}^{k}) = 0\}$ is the new cutting plane for $\mathit{VI}(F, C_{j}^{k})$.
  Increase *k* by one GO TO Step 2.

### Theorem 6

*Let* $F: X \rightarrow\mathbb{R}^{{n}}$ *be strongly monotone on* *X*, *then Algorithm *5 *either stops with a solution of* $\mathit{VI} (F, X )$ *after a finite number of iterations*, *or there exists a subsequence of the infinite sequence* $\{x_{j}^{k}\}$ *that converges to a point* $x ^{*} \in X ^{*}$.

### Proof

*F* is strongly monotone on *X* implies that there exists a constant $N> 0$ such that [22]

$$\max_{y \in X}F(x)^{T}(x - y) \ge N\|x - y \|^{2},\quad\forall x \in X. $$

Let $x_{j}^{*}$ be the unique solution of $\mathit{VI}[F, C _{j}]$ over $C _{j}$ ($j = 1, 2, \dots$). Suppose all boundary points of *X* (if there are any) are in $C _{j}$ ($j = 1, 2, \dots$). Then, if *j* is large enough (say $j > k$), by Lemma 2 we have

$$x_{j}^{*}= x^{*}\quad (j > k). $$

If Algorithm 5 does not stop after a finite number of iterations, then exists an infinite sequence $\{x_{j}\}\subseteq X$ with $x _{j} \in C _{j}$ such that

$$g_{C_{j}}(x_{j}) < \frac{1}{2^{j}}\quad (j = 1, 2, \dots). $$

Hence

$$N\big\| x_{j} - x^{*}\big\| ^{2} = N\big\| x_{j} - x_{j}^{*}\big\| ^{2} \le g_{C_{j}}(x_{j}) = \max_{y \in C_{j}}F(x)^{T}(x_{j} - y) < \frac{1}{2^{j}}\quad(j > k), $$

which implies that $\{x_{j}\}$ is a bounded sequence. Therefore, ∃subsequence of $\{x_{j}\}$, which is convergent to $x ^{**}$ in *X*. Similar to the proof of Theorem 2, $x ^{**}$ is a solution for $\mathit{VI}[F, X]$, and so $x ^{**} = x ^{*}$. □

We notice that, in the proof of Theorem 6, the key condition is that $\{x_{j}\}$ in *X* is a bounded subsequence. Therefore, similarly we have the following theorem.

### Theorem 7

*Let* $F: X \rightarrow\mathbb{R}^{{n}}$ *be strongly pseudomonotone on* *X*, *then Algorithm *5 *either stops with a solution of* $\mathit{VI} (F, X )$ *after a finite number of iterations*, *or there exists a subsequence of the infinite sequence in* *X* *that converges to a point* $x ^{*} \in X$.

Theorems 6 and 7 state that Algorithm 5 can always stop and output an approximate solution after a finite number of iterations.
